# Supplementary material for: Deciphering amyloid fibril molecular maturation through FLIM-phasor analysis of thioflavin T
Source: Biophys Rep (N Y). 2024 Feb 1;4(1):100145. doi: 10.1016/j.bpr.2024.100145 (PMC10884809; doi:10.1016/j.bpr.2024.100145)
Supplement: Document S1. Figures S1 and S2 [file mmc1.pdf]

Biophysical Reports, Volume *4*

Supplemental information

Deciphering amyloid fibril molecular maturation through FLIM-phasor analysis of thioflavin T

Sara Anselmo, Giuseppe Sancataldo, and Valeria Vetri

Deciphering Amyloid Fibril Molecular Maturation through FLIM-Phasor Analysis of Thioflavin T

Sara Anselmo[1,*], Giuseppe Sancataldo[1,] and Valeria Vetri[1]

[1]Dipartimento di Fisica e Chimica – Emilio Segré, Università degli Studi di Palermo, Viale delle Scienze ed. 18, 90128 Palermo, Italy

* Corresponding author:
sara.anselmo@unipa.it

Supporting Material

ThT-stained lysozyme sample after 30 min of incubation

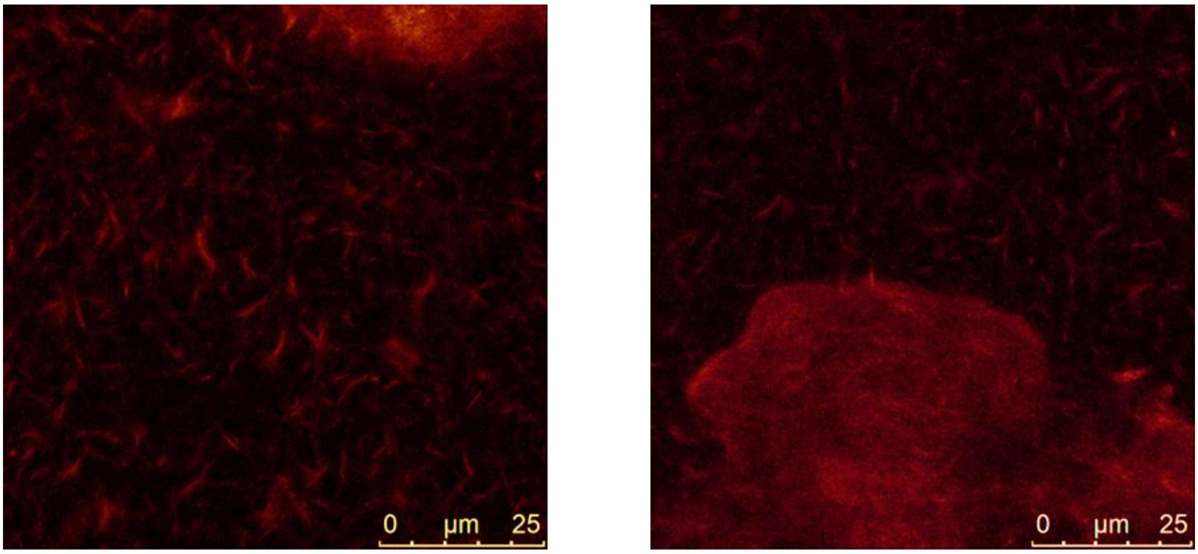

SM.1 1024x1024 pixels representative confocal images of 40 mg/ml HEWL pH 2.0 incubated at 90°C with magnetic stirring at 300 rpm for 30 min and stained with 60 µM ThT (λ_{exc}= 470 nm, emission range 485 nm -600 nm).

Ovoid-shaped lysozyme aggregates formed after 60 miN of incubation

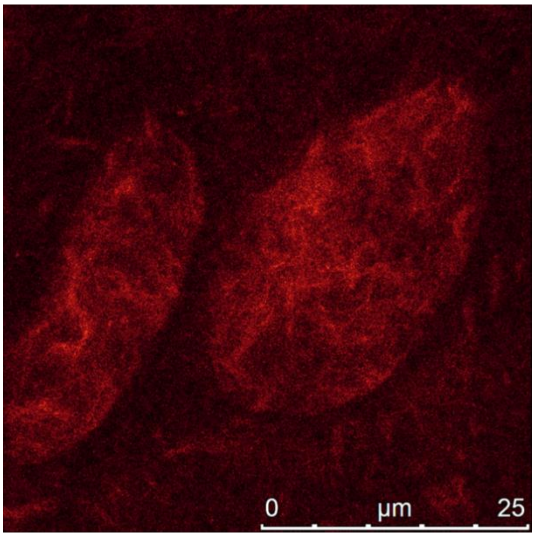

SM.2 1024x1024 pixels representative confocal images of 40 mg/ml HEWL pH 2 incubated at 90°C with magnetic stirring at 300 rpm for 60 min and stained with 60 μM ThT (λ_{exc}= 470 nm, emission range 485 nm -600 nm).
