# Supplementary material for: Deciphering amyloid fibril molecular maturation through FLIM-phasor analysis of thioflavin T
Source: Biophys Rep (N Y). 2024 Feb 1;4(1):100145. doi: 10.1016/j.bpr.2024.100145 (PMC10884809; doi:10.1016/j.bpr.2024.100145)
Supplement: Document S2. Article plus supporting material [file mmc2.pdf]

Biophysical Reports

ARTICLE

Deciphering amyloid fibril molecular maturation through FLIM-phasor analysis of thioflavin T

Sara Anselmo,[1,*] Giuseppe Sancataldo,[1] and Valeria Vetri[1]
[1]Dipartimento di Fisica e Chimica – Emilio Segré, Università degli Studi di Palermo, Palermo, Italy

ABSTRACT The investigation of amyloid fibril formation is paramount for advancing our understanding of neurodegenerative diseases and for exploring potential correlated therapeutic strategies. Moreover, the self-assembling properties of amyloid fibrils show promise for the development of advanced protein-based biomaterials. Among the methods employed to monitor protein aggregation processes, fluorescence has emerged as a powerful tool. Its exceptional sensitivity enables the detection of early-stage aggregation events that are otherwise challenging to observe. This research underscores the pivotal role of fluorescence analysis, particularly in investigating the aggregation processes of hen egg white lysozyme, a model protein extensively studied for insights into amyloid fibril formation. By combining classical spectroscopies with fluorescence microscopy and by exploiting the fluorescence properties (intensity and lifetime) of the thioflavin T, we were able to noninvasively monitor key and complex molecular aspects of the process. Intriguingly, the fluorescence lifetime imaging-phasor analysis of thioflavin T fluorescence lifetime on structures at different stages of aggregation allowed to decipher the complex fluorescence decay behavior, highlighting that their changes rise from the combination of specific binding to amyloid typical cross-β structures and of the rigidity of the molecular environment.

SIGNIFICANCE This research investigates amyloid fibril formation, focusing on hen egg white lysozyme, strategically chosen as a paradigm for amyloid fibril formation. The use of phasor fluorescence lifetime imaging with thioflavin T staining simplifies data analysis, enhancing accessibility by eliminating extensive fitting processes. The approach intuitively highlights structural changes within aggregates, crucial for researchers studying the interplay between structure, function, and properties. The dynamic alteration of thioflavin T quantum yield during aggregation, vital for accurate modeling of amyloid assembly kinetics, is also highlighted. Beyond uncovering hen egg white lysozyme fibril formation aspects, this transformative methodology has broad implications for studying structural changes during aggregate maturation, notably enabling accurate and fast selection of species at the microscale with specific structural properties.

INTRODUCTION

The formation of amyloid structures, associated with significant human disorders such as Alzheimer's, Parkinson's, Creutzfeldt-Jakob disease, and type 2 diabetes, is of paramount interest (1,2). Amyloid fibrils, characterized by highly organized β structures stabilized by a specific hydrogen-bond (H-bond) pattern (3), represent the most stable state for a polypeptide chain (4,5). Although diseases related to amyloid fibrils have attracted major attention, nontoxic and functional amyloid fibrils are also extensively studied and widely characterized. Recent research has explored amyloid structures as potential biomaterials with diverse applications, including conductive nanowires (6,7), sensors (8), absorbent for pollutant removal from water (8–10), scaffold for tissue engineering (11), and so on (8,12). A large number of studies has shown that structurally distinguishable isoforms of amyloid fibrils can form due to differences in inter- or intra-residual interactions and amyloid aggregates can adopt distinct conformations at the molecular level, giving rise to multiple isoforms with unique structural characteristics. Amyloid aggregates can also adopt distinct conformations at the molecular level, leading to diverse 3D arrangements such as particulates (13), spherulites (13), or hydrogels (11).

Submitted December 5, 2023, and accepted for publication January 25, 2024.

*Correspondence: sara.anselmo@unipa.it

Editor: Jorg Enderlein.

https://doi.org/10.1016/j.bpr.2024.100145

© 2024 The Author(s).

This is an open access article under the CC BY license (http://creativecommons.org/licenses/by/4.0/).

The exceptional properties of amyloid structures, such as stability, rigidity, chemical properties, high aspect ratio, and conductivity, stem from regular H-bond patterns that stabilize their molecular arrangement. Variations at the molecular level, occurring during the maturation (14,15) process, contribute to the complexity and heterogeneity observed in amyloid structures, with different molecular arrangements and structural organization across various length scales underlying their multifunctionality (9,10,12).

In this study, the fluorescence properties of thioflavin T (ThT), coupled with confocal fluorescence microscopy, fluorescence lifetime imaging microscopy (FLIM), and classical spectroscopic tools, were used to monitor hen egg white lysozyme (HEWL) amyloid and formation of a gel-like structure, particularly focusing on the variation occurring at the molecular level. HEWL is a model protein widely used to explore many aspects related to protein structure, folding, and aggregation, in particular for amyloid growth studies (16–22). Many studies focus on HEWL supramolecular assembly under high temperature and acidic pH conditions (18,20,22–25) since, under these conditions, aggregates display key amyloid properties such as β sheet-rich structure, fibrillar morphology, and seeding abilities ranging from small oligomers to large amyloid fibrils and fibrillar hydrogels (26,27). Moreover, due to its large availability and high solubility, HEWL is an excellent choice for the production of fibrillar protein hydrogels with various applications and it was used in the development of new protein-based materials (23,26,28–30) and in templating metal nanoparticles to build aerogel (28). In this context, the fine-tuning of the material properties is strictly related to the knowledge of the detailed molecular structures, which can be regulated by controlling solution conditions or selecting different maturation points (31).

The focus of this work is the analysis of HEWL supramolecular assembly from fibrils formation to the hydrogel state combining different spectral observables in a time-resolved fashion with phasor-FLIM analysis of ThT-stained samples. In the last 50 years, ThT has emerged as a valuable tool in the study of protein aggregation and amyloid fibril formation (32–34). The fluorescence emission intensity signal of this chromophore in an aqueous environment is vanishing due to the presence of highly efficient internal nonradiative channels. However, there is a remarkable increase in signal intensity when amyloid structures are present (35–39). Its increased emission intensity upon binding to amyloid fibrils is due to the inhibition of the rotation around the C–C bond, which causes the electronic wave function in the excited state to change adiabatically from an emissive LE state to a dark (nonradiative) CT state (40–42). Many studies have also shown that ThT fluorescence signal grows in highly viscous media (43) and in the presence of different confining systems, from DNA to porous silica (44). Notwithstanding this, the exquisite sensitivity of ThT to the formation of amyloid fibrils (32,41,45–47) and also to their structural peculiarities (48) is largely assessed. Importantly, ThT quantum yield was found to relate to the viscosity (43,49), the polarity (39), the geometry of the surrounding environment and morphology of the amyloid (48,50). It is not surprising, then, that fluorescence decays of ThT could exhibit distinct characteristics when the dye is bound to proteins at different stages of aggregation kinetics, providing valuable insights into the evolution and maturation of aggregates (46,51). Noteworthy, the fluorescence intensity of ThT may be influenced by both the affinity and accessibility of its binding sites. Accessibility depends on the particular geometric arrangement of the aggregate microstructure and the surrounding solvent conditions and regulates the number of bound molecules. On the other hand, the affinity primarily relies on the structural specificity of the binding sites, often associated with the presence of β sheet structures. For these reasons, opting for the examination of ThT lifetime would be more advantageous for gaining precious information. However, this is frequently challenging and complicated due to the requirement for a clear-cut model. In many studies, a multi-component analysis (involving two or three components) has been applied to ThT fluorescence lifetime for the analysis of various systems (9,31,45,46,52–54). Across all these studies, a common finding seems to suggest that a sub-nanosecond component can be associated with environmental rigidity in the surrounding of the molecular rotor, whereas longer lifetime components relate to the specific binding sites, namely structural peculiarities of cross-beta structures. Here, the phasor approach to analyze FLIM data of ThT-stained samples simplifies fluorescence lifetime representation, providing a powerful method (55) for extracting information on aggregated molecular structures during HEWL fibril maturation. This allows visualization of structural differences at various aggregation stages, enabling a multi-faceted investigation of fibril formation. Combining spectroscopic analyses with high-resolution imaging offers a holistic view of the aggregation phenomenon, providing insights into structural dynamics and spatial distribution of fibrils. Phasor analysis of ThT-stained protein samples proves a rapid, robust, and straightforward method for characterizing amyloid fibril structure at the submicrometer scale, enabling real-time analysis and identification of species during aggregate formation, even in highly heterogeneous samples.

MATERIALS AND METHODS

Chemicals and reagents

Lysozyme from hen egg white (≥90%, L6876) and ThT (≥ 65%, T3516) were purchased from Sigma-Aldrich. Hydrochloric acid standard solution (343102) was purchased from Fluka.

Sample preparation

HEWL (40 mg/mL) was dissolved in Milli-Q water acidified to pH 2.0 with HCl and filtered through 0.45-μm syringe filters before thermal treatment. A sealed glass bottle containing 6 mL of the protein solution was placed in a glycerol bath at 90°C under stirring at 300 rpm for up to 2.5 h (18). Aliquots of the protein solutions were collected at different incubation time points and quenched in an ice-water mixture to block aggregation processes. This procedure enables us to choose various time points, with a maximum error of 1.0 min for each selected point. Data are shown as representative of several analogous experiments performed in the same conditions.

Circular dichroism

Circular dichroism (CD) spectra of 1:40 diluted HEWL pH 2.0 before and after heating at 90°C at different incubation stages were recorded with a Jasco J-715 spectropolarimeter in the far-UV region (194–260 nm) using quartz cuvettes with a path length of 0.2 mm. For each spectrum, four accumulations were acquired, with data interval 0.2 nm, bandwidth 1 nm, and scan speed 50 nm/min. All spectra were acquired at room temperature.

Fourier transform infrared spectroscopy

Measurements were carried out at room temperature with a Bruker Vertex 70 spectrometer equipped with a doped triglycine sulfate detector, in a sample compartment under continuum purging and in N_2 dry atmosphere to reduce water vapor. Native HEWL was dissolved in D_2O and the aggregated HEWL samples were washed several times in D_2O, to remove water, using 10-kDa MWCO spin filters (25°C, 14,000 rpm, 15-min cycles). The filtrate did not contain measurable amounts of protein. Washed samples, identically treated, were placed between two CaF_2 windows separated by a 50 μM Teflon spacer. Each spectrum is an average of 256 scans in the 400–7000 cm^{-1} range with a spectral resolution of 2 cm^{-1}. Sample and solvent absorption spectra were calculated with respect to the spectrum of the empty cell.

Confocal microscopy and FLIM

Each sample was stained with 60 μM ThT, and 250 μL of samples were placed in microscope-chambered slides and imaged at 1024 × 1024 pixel resolution, using a Leica TCS SP5 confocal laser scanning microscope and a 63×/1.4 oil objective (Leica Microsystems, Germany). In these conditions, low protein/dye ratio rules out fluorescence quenching phenomena (47). ThT was excited using $\lambda_{ex} = 470$ nm (white light laser, repetition rate 80 MHz (Leica Microsystem, Germany)) and the emission signal was collected in the range 485–600 nm. By employing a repetition rate of 80 MHz, we transmit pulses at intervals of 12.5 ns. In this way, we avoid potential critical issues associated with laser repetition rates, much faster than the fluorescence decay completion (56–59). Fluorescence lifetime imaging measurements were acquired in the time domain by means of a picoHarp 300 standalone TCSPC module (Picoquant), and 256 × 256 pixel images were obtained at a scanning frequency of 400 Hz (pixel size 0.19 × 0.19 μm) using the aforementioned laser parameters.

FLIM-phasor plot analysis and interpretation

The phasor analysis, described by Digman et al. (60), was used for FLIM data. The values of the sine-cosine transforms measured in every pixel of the image are represented in a polar plot as a two-dimensional histogram (phasor plot). Each pixel of the image gives a point in the phasor plot. In this representation, all possible single exponential decays lie on the "universal circle" defined as a semicircle, with radius ½, going from point (0,0), corresponding to $\tau = \infty$, to point (1, 0), corresponding to $\tau = 0$. Instead, complex decays are represented by phasors within the universal circle. Importantly, given that the phasors follow the vector algebra, it is possible to geometrically resolve the fractions of two fluorescent species (in the simplest case) by the lever rule of vector additions. Indeed, the linear combination of two single exponential decays components generates phasors within the universal circle, which lie on a straight line joining the phasors of the two single components. The contribution of one selected single component to the lifetime is proportional to the distance of the other single component from it. In simpler terms, as the fractional intensity contribution increases, the image phasor point moves closer to the corresponding contributing species phasor. The phasor plot is also used in a reciprocal mode in which each (occupied) point of the phasor plot can be selected using colored cursors and mapped to a pixel of the image with the same color of the cursors. In this way, the so-called lifetime maps are obtained. FLIM data were processed by the SimFCS4 software (Laboratory for Fluorescence Dynamics, University of California, Irvine, CA, available at www.lfd.uci.edu) and FLIM calibration of the system was performed by measuring the known lifetime of the fluorescein in aqueous solution at pH 8 that is a single exponential of 4.0 ns (61).

RESULTS

HEWL fibril formation was induced by incubating 40 mg/mL protein in HCl solution at pH 2.0 under magnetic stirring at 90°C. Aggregation was quenched at different time points in an ice/water bath. The sample was imaged using fluorescence confocal microscopy after staining with 60 μM ThT. In Fig. 1 *a*, we report 1024 × 1024 pixels representative fluorescence confocal microscopy images of the samples measured after 30, 37, 60, and 150 min of incubation. Fig. 1 *b* reports the average intensity fluorescence of ThT (five measurements on images of the same size for identically treated samples) as a function of the incubation time. Control measurements on native HEWL dissolved in solution pH 2.0, stained with ThT under the same conditions, show no significant fluorescence signal.

Data in Fig. 1, as expected, reveal the progressive growth of the size and changes in the fibrils' morphology. Elongated bright micrometer-scale aggregates, which appear as fibril bundles, are evident since the initial phases. As incubation time progresses, a net-like (gel-like) organization emerges as is evident from the images. The growth of ThT fluorescence intensity of the whole images and at single-aggregate

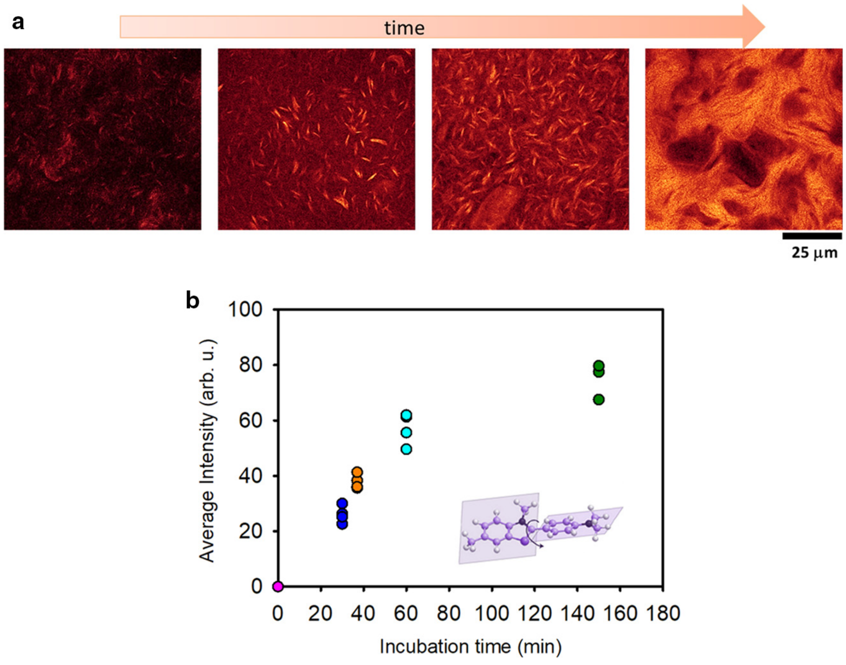

FIGURE. 1 ThT fluorescence intensity. (*a*) 1024 × 1024 pixels representative confocal fluorescence microscopy images of 40 mg/mL HEWL pH 2.0 incubated at 90°C with magnetic stirring at 300 rpm for 30, 37, 60, and 150 min stained with 60 μM ThT (λ_{exc} = 470 nm, emission range 485–600 nm). (*b*) Average ThT fluorescence intensity measured in four different images, of the same size, acquired for each sample incubated at different times. ThT molecular structure is also reported.

level is also detected as a function of time. The time evolution of fluorescence intensity is reported in Fig. 1 *b*. As described above, ThT is one of the most common tools used to analyze the formation of amyloid structures. It shows high selectivity for amyloid fibrils and, upon binding to amyloid aggregates, it shows a bright fluorescence emission in the visible region (42,62). The increase in ThT fluorescence intensity is believed to be associated with the abundance of fibrillar structures in the solution, making it an effective and sensitive reporter (63). Accordingly, measurements in Fig. 1 *a* and *b* show that the increase of ThT fluorescence intensity is attributed to the growth of the number and the size of the aggregated species in the sample but also to the signal enhancement at the single-aggregate level. We attribute this to changes in the protein structure, resulting in increased affinity between the dye and the aggregates, and/or changes in the quantum yield of the dye induced by the modified environment. Specifically, it is clear that HEWL sample, treated for 30 min, forms ThT-positive fibrillar structures. There is significant heterogeneity characterized by the coexistence of nonfluorescent regions (black areas), small fibrils, and large clusters of fibrils (see Fig. S1). Extending the incubation time to 37 min results in a greater abundance of fibrils, approximately 6 μm in length. After a 37-min treatment, the previously observed dark areas in the sample are no longer present. Notably, a uniform fluorescence with a diffuse background is now visible, indicating the presence of small structures with size below spatial resolution, which show a positive response to ThT. The sample incubated for 60 min is entirely populated by aggregates that exhibit strong ThT fluorescence signal with distinct and well-defined fibrils. Additionally, large ovoidal aggregates are also visible, containing clearly identifiable individual fibril structures within them (see Fig. S2). After 150 min of heating and continuous agitation, the individual aggregates are no longer distinguishable, and thin fibrils are organized in a gel-like structure.

In line with the literature, amyloid fibrils are formed in the present conditions, which readily further assemble to form an amyloid-like hydrogel (26,27).

To gain information on the average molecular arrangement of HEWL amyloid structures at different time points during the observed supramolecular assembly, CD and Fourier transform infrared (FTIR) bulk measurements on the same samples were performed. These spectroscopic methods are widely used for determining the secondary structure of proteins and peptides enabling the monitoring of structural changes (64–67). Both of these techniques present a remarkable sensitivity to the molecular arrangement of protein aggregates (13) and allow the analysis of protein samples in different concentration regimes.

Fig. 2 *a* shows the far-UV CD spectra of the samples diluted 1:40 after 30, 37, 60, and 150 min of heating treatment at 90°C under magnetic stirring; the spectrum of the native protein in the same solution conditions is also reported for comparison. Significant

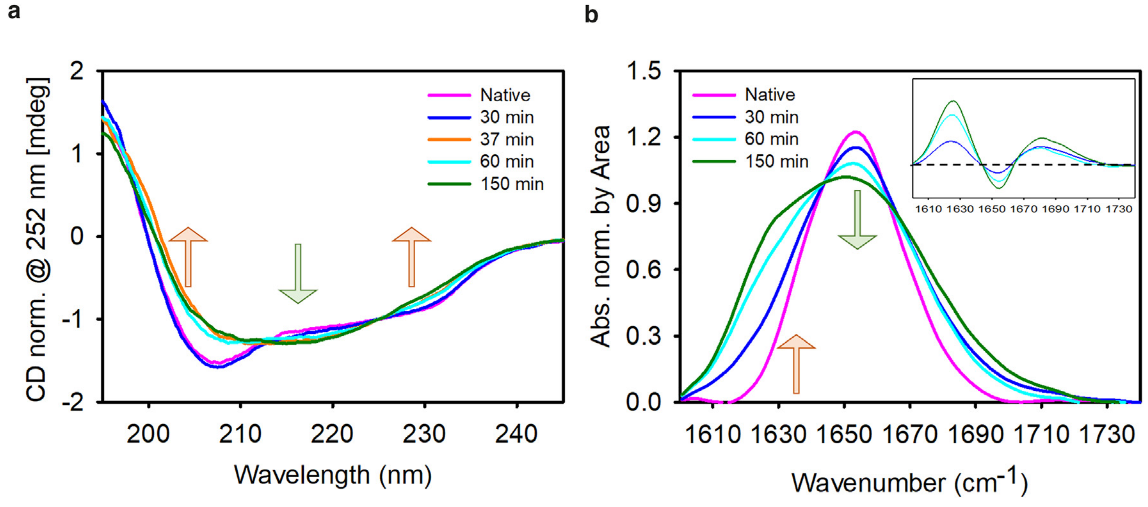

FIGURE. 2 Spectroscopic analysis. (*a*) Far-UV (194–260 nm) CD spectra, normalized at 252 nm, of HEWL after 30, 37, 60, and 150 min of incubation at pH 2.0 and 90°C under magnetic stirring at 300 rpm in comparison with the CD spectrum of the native protein in the same solution conditions (*pink line*). The measurements were performed in triplicates. (*b*) FTIR spectra in the amide I′ region (1735–1580 cm^{-1}) of HEWL after 30, 60, and 150 min of incubation at pH 2.0 and 90°C under magnetic stirring at 300 rpm in comparison with the FTIR spectrum of native protein in the same solution conditions (*pink line*). In the insert, the differential spectra are shown. All these samples are dissolved in D_2O (see section "materials and methods").

changes in spectral shape are observed during the supramolecular assembly. The native HEWL sample (pink curve), in line with the literature (18), is characterized by a CD spectrum with a sharp minimum at 208 nm and a broad one in the 220- to 230-nm region, which are characteristics of the native structure of HEWL, which comprises both α helices and β strands. As shown in Fig. 2 *a*, comparing the spectrum of the native HEWL with that of the sample heated for 30 min, the bulk CD measurements fail to highlight the differences between the two samples found by confocal microscopy measurements. Although aggregates are visible in Fig. 1 *a*, at this stage they could represent a minor fraction of the sample, with respect to native or native-like protein structure, not giving rise to a detectable signal in the presented experimental conditions. A significant spectral change is observed after 37 min of incubation, clearly indicating that significant structural changes occurred. Specifically, the decrease in the ellipticity of the peak at 208 nm, accompanied by an increase in the ellipticity of the peak at about 217 nm, confirms the hypothesis that the sample undergoes a transition from its initial native structure to intermolecular β sheet structures (68,69). Changes measured at 230 nm were previously attributed (for equine lysozyme) to modifications in aromatic residues packing during amyloid fibrils formation (68). Under these conditions, the fluorescence microscopy images at the same time point reveal an increased presence of micrometer-scale bright aggregate structures alongside a diffuse fluorescence that was not previously observed. The CD spectra of the samples at longer time points does not show significant differences, notwithstanding critical morphological and intensity differences observed using fluorescence microscopy. Fig. 2 *b* shows FTIR spectra, normalized by the area in the amide I′ region (1575 cm^{-1}–1710 cm^{-1}), of native HEWL and after 30, 60, and 150 min of incubation. The spectrum of native HEWL in D_2O is also reported and resembles the one widely reported in the literature for HEWL with a peak centered at around 1654 cm^{-1}. Differential spectra with respect to the native structure are reported in the inset. As can be seen, the spectrum measured after 30 min of thermal/stirring treatment is characterized by a decrease in the intensity of the native peak at 1654 cm^{-1} and a broadening of the band with respect to the spectrum of the native HEWL. The samples incubated for longer time points show a progressive decrease of the peak at 1654 cm^{-1} accompanied by the growth of the peak at about 1620 cm^{-1}, which is assigned to parallel intermolecular β sheets and considered a hallmark of amyloid structures (70,71). A parallel growth of the peak centered at 1680 cm^{-1}, assigned to antiparallel intermolecular β sheets, is also observed. Over time, the absorption peak at around 1620 cm^{-1} grows, broadening its width toward shorter wavenumbers. This could be attributed to increased intermolecular H-bond strength or increased number of β sheets (72,73), in line with the increasing ThT fluorescence intensity measured and reported in Fig. 1. Although bulk measurements only provide averaged information, where the contribution of less numerous but still relevant

species may be underestimated, data in Fig. 2 confirm a direct correlation between an extended incubation time and an increased presence of intermolecular β-structure content that evolves in parallel with ThT intensity growth.

In summary, the incubation at high temperature and under stirring leads to the development of HEWL fibrils. The size, shape, and structure of the resulting aggregate species undergo changes over time, as shown by fluorescence microscopy, illustrating the gradual formation of micrometer-scale fibrils that progressively assemble into larger structures, resulting in a dense network in the final stages. CD measurements on the samples obtained by incubation at 30 and 37 min suggest that small, under-resolution, ThT-positive species are formed in the earlier stages. In line with the literature, these species may constitute seeds involved in heterogeneous nucleation as also suggested by the concave shape of measured kinetics (74). We wish to note that the detailed analysis of nucleation mechanisms is out of the scope of this manuscript and widely assessed in literature for HEWL in these conditions (20). Importantly, a variety of species were observed, with those testing positive for ThT exhibiting a consistent fluorescence pattern that appears to be correlated with their individual sizes and morphologies. The average intensity captured within a single image, akin to what would be derived from a bulk measurement, demonstrates an upward trend owing to the growth of aggregate numbers in the solution and of the brightness of individual species over time. This specific feature may be due to the increase in the density of ThT-binding sites within the single voxel and/or to changes in ThT quantum yield related to variations in the environment surrounding the binding sites.

In previous studies, it was shown that a certain correlation exists between ThT fluorescence lifetime and the detailed molecular architecture of the stained amyloid structures (9,31,36,52) and that distinct architectures in amyloid fibrils lead to different lifetimes of ThT fluorescence (31,46). Indeed, as previously mentioned, the fluorescence-sensing properties of ThT are influenced by the affinity between the dye and the binding sites, as well as the interaction of ThT with its surroundings (43,51). Measurements of ThT fluorescence lifetime provide distinct indications of various binding modes and differences in amyloid structures, offering a notable advantage over assessing ThT intensity alone. Importantly, fluorescence lifetime measurements are not contingent on the concentration of chromophores, suggesting independence from the number of molecules occupying the binding sites. This independence enhances the capability to analyze amyloid structures comprehensively.

In Fig. 3, we report the FLIM analysis of measurements acquired on the HEWL samples (40 mg/mL) incubated at 90°C for 30, 37, 60, and 150 min under stirring after staining with 60 μM ThT. The phasor approach presented here to analyze FLIM data has the potential of simplifying the analysis, avoiding some of the problems of the exponential analysis and providing a graphical global view of the processes affecting the fluorescence decay occurring at each pixel (56,60).

In Fig. 3 *a–d*, 256 $\times$ 256 pixels ThT fluorescence intensity maps of the samples incubated for the different interval times are shown. In Fig. 3 *e–h*, we report the phasor maps in which each pixel is colored according to the colored cursors used to select the four lifetime distributions shown in the phasor plot (Fig. 3 *i*). This analysis simplifies the identification of ThT fluorescence decays in each pixel. Moreover,

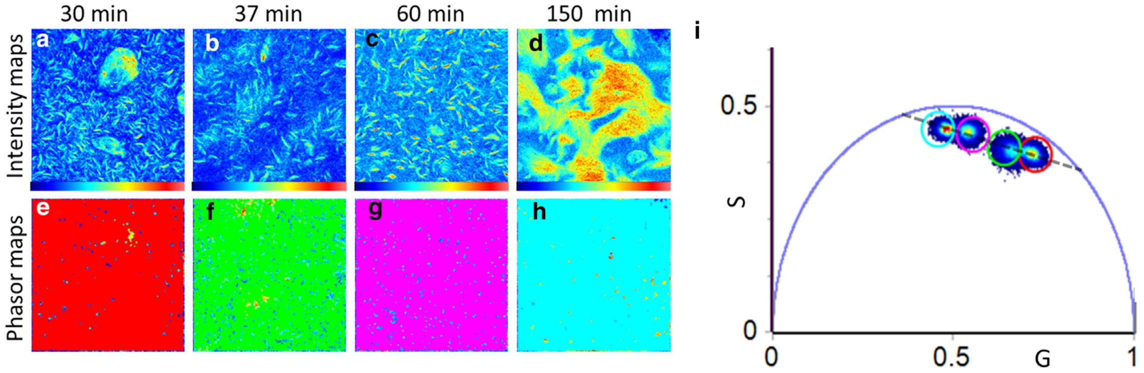

FIGURE. 3 Phasor analysis. Phasor analysis of FLIM measurements on HEWL samples (40 mg/mL) incubated at 90°C under 300 rpm stirring for 30, 37, 60, and 150 min and stained with 60 μM ThT. The signal is acquired under laser excitation at 470 nm, in the range 485–600 nm. (*a,b,c,d*) Intensity maps and (*e,f,g,h*) the lifetime maps, corresponding to (*a,b,c,d*) measurements, colored according to the color circles used to highlight the lifetime clouds in the phasor plot (*i*). Red pixels are related to shorter lifetimes. The progressively increasing lifetimes are mapped using green, pink, and cyan colors.

measured lifetime distributions lie inside the universal circle, indicating that ThT lifetimes in these conditions are characterized by nonsingle exponential decay. In line with previous observations, findings suggest that the lifetime of ThT exhibits nonsingle exponential decay patterns, representing mixtures of different components (31,53).

Our results reveal that the average of ThT increases as a function of time as aggregation proceeds. In particular, referring to Fig. 3, the lifetime distribution moves from the position highlighted with the red cursor to the one marked with the cyan cursor going from 30 to 150 min of incubation. Additionally, this analysis emphasizes a remarkable level of homogeneity of ThT lifetime within each sample despite the large heterogeneity of species with different size and morphology.

In Fig. 4, a comprehensive quantitative analysis is presented, utilizing the decomposition of the phasor plot data with a focus on two principal lifetime components. It is crucial to note that a similar analysis has previously been conducted in various systems, as referenced in the literature (31,51,75). This analytical approach represents the simplest model employed to elucidate the ThT fluorescence decay under the specified conditions. The utilization of two principal lifetime components enhances the ThT behavior analysis, allowing for a deeper understanding of the intricate fluorescence dynamics associated with these conditions. Although the existence of additional components cannot be excluded (76), this description allows the straightforward interpretation of fluorescence signal, intuitively spotlighting structural changes within aggregates. In addition, we note that confirming a detailed model is often less critical compared to fitting procedures. This is because the information is embedded in the heuristic phasor clouds' "trajectory," which is determined through the Fourier transformation of fluorescence decay.

In the FLIM analysis reported in Fig. 4, we used two mono-exponential components characterized by $\tau = 0.7$ ns (red cursor) and $\tau = 2.7$ ns (green cursor), identified via the intersection of the straight line, passing through all the lifetime distribution clouds in the phasor plot in Fig. 3. These values are significantly higher when compared to those observed in water, where the lifetime of ThT falls within the picosecond range (43,77).

Similar to previous studies on different proteins in different solution conditions, it is possible to attribute the fastest decay (0.7 ns) to less-specific binding sites where ThT fluorescence is triggered by increased environmental viscosity. Slower decay (2.7 ns) can be attributed to more-specific interactions between ThT and intermolecular β structures that add more constraints and less flexibility to the ThT-binding site. Data can be analyzed using the two-components model

$$I(t) = A_1 e^{-\frac{t}{\tau_1}} + A_2 e^{-\frac{t}{\tau_2}}$$

where A_1 and A_2 are the amplitude of the single exponential decays with τ_1 and τ_2 representing the fast and slow average lifetimes. In the phasor plot, the distance between each point of the cloud and the single-exponential phasor is related to the fractional intensities (F1 and F2) of each component which are proportional to A_1 and A_2. In Fig. 4 *a–d*, measurements reported in Fig 3 *a–d* are shown in false colors according to the F_2 fraction of the τ_2 component. The scale goes from red (pure fast component at $\tau_1 = 0.7$ ns) to blue (pure slow component at $\tau_2 = 2.7$ ns). The fastest decay is dominant at the earlier stages of aggregation (30 min, $F_2 = 0.25$) this seems to follow minor changes observed in bulk at the secondary structure level (Fig. 2). The average fraction F_2 grows, increasing the incubation time from $F_2 = 0.38$ (after 37 min) to $F_2 = 0.85$ (after 150 min), passing through $F_2 = 0.77$ (after 60 min), paralleling the growth of intermolecular β sheet reflected by CD and FTIR spectra.

At the earlier stages of aggregation, the formation of protein assemblies results in the increase of the microviscosity (or solution-free volume) due to local growth of protein concentration in the supramolecular

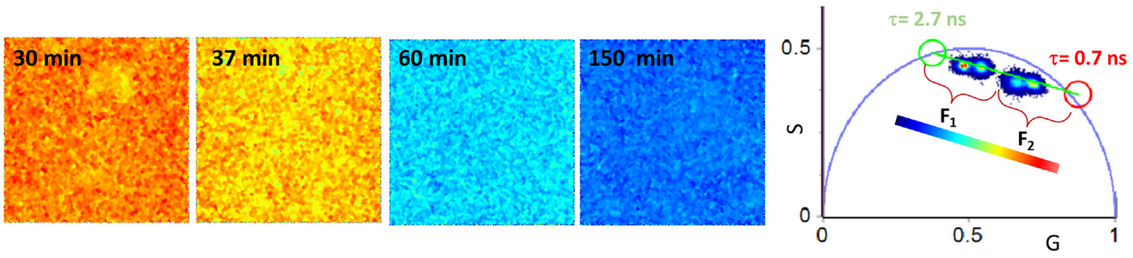

FIGURE. 4 Data analysis. Data analysis of the measurements reported in Fig. 3 performed using a double exponential decay whose principal components are $\tau_1 = 0.7$ ns (*red*) and $\tau_2 = 2.7$ ns (*green*) identified drawing a straight line interpolating the two lifetime distributions clearly evident in the phasor plot. The lifetime fraction maps for the FLIM images are colored according to the fractional contribution of the slower component (F_2).

assemblies. This could be accompanied by the occurrence of low-affinity binding modes of molecular rotors for different constituents of the aggregation mixture (such as monomers, oligomers, and fibrils) also affecting viscosity around the dye (78). Progressive increase of lifetime can be related to β-structure maturation resulting in stronger H-bonds and/or longer β chains within the aggregates.

In summary, the presented study shows that, under heating and stirring, HEWL molecules undergo a series of dynamic events that lead to their mutual interaction to assemble into fibrils. As the incubation time increases, the number of fibrils in the solution continues to rise, indicating ongoing aggregation. Eventually, these fibrils further aggregate with each other, giving rise to a massive quantity of larger species and finally to a gel. The observed increase in emission intensity and lifetime of the ThT probe have potentially been attributed both to the rise in intermolecular β structures characterized by tight H-bonds and also to the heightened viscosity of the surrounding environment. Interestingly, the increased ThT lifetime parallels the growth and the increase in strength of intermolecular structures highlighted by FTIR; fluorescence lifetime maps allow a qualitative visualization of the increase in β structures during aggregation.

DISCUSSION

The possibility of obtaining molecular-level information on amyloid aggregate structures is of great interest, as it has significant implications in various research fields. Understanding these structures can shed light on molecular mechanisms involved in neurodegenerative pathologies, can aid in controlling the immunogenic risk of protein drug products, and can offer opportunities to design new protein-based materials with controllable properties.

In this study, we analyzed the aggregation of HEWL induced by thermal conditions in acidic environments with stirring. We identified a straightforward, homogeneous amyloid formation pathway, showcasing structurally uniform amyloid species at each observed time point despite morphological inhomogeneity. By employing fluorescence microscopy methods, CD, and FTIR spectroscopy, we tracked the progression of structural changes occurring during thermal aggregation.

Microscopy techniques were employed to analyze the fluorescence signal of ThT, enabling the direct visualization and real-time monitoring of aggregate formation at the micrometer scale. This approach facilitated the identification of distinct aggregate populations at various maturation stages. Notably, we observed that changes in the secondary structure toward the formation of intermolecular β sheets were concurrent with the increase in ThT fluorescence lifetime. Interestingly, results show that, at the same maturation stage, the dye experienced similar environments despite differences in the morphology and size of the observed species, likely due to sample stirring, which is known to minimize the effects of heterogeneous nucleation phenomena.

The analyzed systems highlighted that FLIM-phasor analysis of ThT-stained protein aggregates provides an accessible and immediate method to capture details on sample morphology and molecular structure at submicrometer resolution. In particular, it allows the progressive enhancement of intermolecular β sheet strength/content to be highlighted.

Phasor analysis offered a straightforward and rapid approach for analyzing FLIM data without requiring specific models that could bias data interpretation. In the presented conditions, we had the possibility to easily assess that amyloid growth regularly proceeds over time: the sample structural uniformity during gel formation and the ease of controlling it may simplify the selection of building blocks with diverse properties when designing amyloid-based materials.

Phasor-FLIM analysis revealed hidden information within the complex ThT decays, providing insights that are challenging to obtain using bulk methods and affirming the valuable sensitivity of fluorescence techniques. The analysis of ThT fluorescence lifetimes during HEWL fibrillogenesis revealed that the decays followed individual distributions lying on a straight line connecting two distinct lifetime values (0.7 ± 0.2 ns and 2.7 ± 0.2 ns), consistent with those described for other amyloidogenic proteins under varying conditions (31,45,46). This allowed us to describe ThT fluorescence decays in terms of the superimposition of two primary effects: the viscosity of the environment, associated with the sub-nanosecond component, and the ThT binding specificity to the intermolecular β structures in amyloids, represented by the longer lifetime component.

To characterize the structural properties of amyloids, we opted for simpler models that describe the trajectory of phasor clouds within the phasor plot, solely determined through the Fourier transformation of measured fluorescence decays. Through this approach, we demonstrate the ability to track the growth of intermolecular β sheets during HEWL fibrillogenesis. This is achieved by monitoring the shift of the ThT fluorescence lifetime distribution along the line, transitioning from a position closer to the sub-nanosecond component toward the longer lifetime component. This trajectory resembled that observed during

the maturation of alpha-lactalbumin particulates (79) and the formation of insulin fibril (31). Intriguingly, amyloid-like superstructures (spherulites) arising from insulin supramolecular assembly, when stained with ThT, exhibited a broad fluorescence lifetime distribution, appearing as a cloud of points extended along the same straight line in the phasor plot (within the instrumental time resolution sensitivity) going from low to high β-aggregate structure content. This observation unveiled significant aggregate-to-aggregate structural diversity and variability within the same aggregate. Furthermore, the coacervation of Pvfp-5β leading to the formation of amyloid fibrils displayed analogous behavior, with early-stage protein droplets showing ThT fluorescence due to increased environmental viscosity and fluorescence lifetime dominated by the sub-nanosecond component (53).

In these instances (31,53), the longer lifetime components along the line characterized ThT decay within structures with a higher or stronger content of intermolecular β structures.

Taken together, these experimental results support the notion that analyzing ThT fluorescence lifetime through the phasor approach provides previously unreported information about the properties of this dye and offers a straightforward means to analyze the molecular structure of protein aggregates. This is summarized in Fig. 5, which illustrates the shift in the ThT lifetime distribution within the phasor diagram due to fibril maturation. ThT signal at the beginning of the aggregation processes is mainly attributable to increased environmental rigidity with nonradiative decay due to ThT rotation. As the aggregation process evolves and maturation of the aggregates occurs, intermolecular β sheet content increases and stronger H-bonds and/or longer β chains provide specific binding sites that add more constraints and less flexibility to the ThT-binding sites. This results in an increase of the fluorescence lifetime causing a shift along a line connecting two single lifetime distributions corresponding to individual components in the phasor plot.

The phasor analysis allowed us to propose a simple model to interpret the significance of these decay times. This capability is crucial as it enables the discrimination of structurally distinct species within aggregated samples and facilitates tracking the temporal evolution of protein assembly processes into amyloid structures. By understanding the dynamics of fluorescence lifetimes and their correlation with specific structural changes, researchers will gain valuable insights into the mechanisms and kinetics of amyloid formation. Such knowledge holds promise for various fields, including biophysics, biochemistry, and biomedical research, where studying amyloid structures is paramount.

SUPPORTING MATERIAL

Supporting material can be found online at https://doi.org/10.1016/j.bpr.2024.100145.

AUTHOR CONTRIBUTIONS

S.A., conceptualization, formal analysis, investigation, methodology, visualization, and writing; G.S., conceptualization, methodology, and writing – review & editing; V.V., conceptualization, funding acquisition, methodology, project administration, resources, and writing – review & editing.

ACKNOWLEDGMENTS

Funding: financial support from the PNR 2015–2020, 4Frailty—ARS01_00345 and Unipa FFR 2023.

DECLARATION OF INTERESTS

The authors declare no competing interests

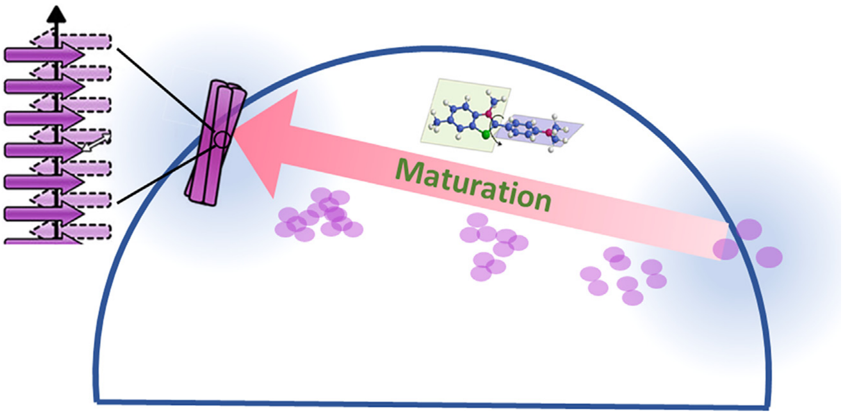

FIGURE. 5 The scheme illustrates the pathway of amyloid structure maturation for amyloid species stained with ThT. Phasors heuristically to describe a linear trajectory connecting the characteristic fluorescence lifetime single exponential components (0.7 ± 0.2 ns and 2.7 ± 0.2 ns). Although alternative complex multicomponents models could be identified, the shift of the phasor cloud along this line well describes the transition from dense structures with low intermolecular β sheet contents to mature amyloid structures characterized by higher content of intermolecular β structures.

REFERENCES

1. Dobson, C. M. 2017. The Amyloid Phenomenon and Its Links with Human Disease. *Cold Spring Harbor Perspect. Biol.* 9:a023648. https://doi.org/10.1101/CSHPERSPECT.A023648.
2. Selkoe, D. J. 2003. Folding proteins in fatal ways. *Nature.* 426:900–990. https://doi.org/10.1038/NATURE02264.
3. Sunde, M. 1997. *In* chemistry CBA in protein. The structure of amyloid fibrils by electron microscopy and X-ray diffraction. Elsevier. https://doi.org/10.1016/S0065-3233(08)60320-4.
4. Thirumalai, D., and G. Reddy. 2011. Protein thermodynamics: Are native proteins metastable? *Nat. Chem.* 3:910–911. https://doi.org/10.1038/NCHEM.1207.
5. Baldwin, A. J., T. P. J. Knowles, ..., C. M. Dobson. 2011. Metastability of native proteins and the phenomenon of amyloid formation. *J. Am. Chem. Soc.* 133:14160–14163. https://doi.org/10.1021/JA2017703.
6. Scheibel, T., R. Parthasarathy, ..., S. L. Lindquist. 2003. Conducting nanowires built by controlled self-assembly of amyloid fibers and selective metal deposition. *Proc. Natl. Acad. Sci. USA.* 100:4527–4532. https://doi.org/10.1073/PNAS.0431081100.
7. Elfwing, A., F. G. Bäcklund, ..., N. Solin. 2015. Protein nanowires with conductive properties. *J. Mater. Chem. C Mater.* 3:6499–6504. https://doi.org/10.1039/C5TC00896D.
8. Bolisetty, S., and R. Mezzenga. 2016. Amyloid–carbon hybrid membranes for universal water purification. *Nat. Nanotechnol.* 11:365–371. https://doi.org/10.1038/nnano.2015.310.
9. Anselmo, S., T. Avola, ..., A. Pettignano. 2023. Sustainable soy protein microsponges for efficient removal of lead (II) from aqueous environments. *Int. J. Biol. Macromol.* 239:124276. https://doi.org/10.1016/j.ijbiomac.2023.124276.
10. Anselmo, S., S. Cataldo, ..., V. Vetri. 2022. Lead(II) ions adsorption onto amyloid particulates: An in depth study. *J. Colloid Interface Sci.* 610:347–358. https://doi.org/10.1016/j.jcis.2021.11.184.
11. Jacob, R. S., D. Ghosh, ..., S. K. Maji. 2015. Self healing hydrogels composed of amyloid nano fibrils for cell culture and stem cell differentiation. *Biomaterials.* 54:97–105. https://doi.org/10.1016/J.BIOMATERIALS.2015.03.002.
12. Knowles, T. P. J., and R. Mezzenga. 2016. Amyloid Fibrils as Building Blocks for Natural and Artificial Functional Materials. *Adv. Mater.* 28:6546–6561. https://doi.org/10.1002/ADMA.201505961.
13. Vetri, V., and V. Foderà. 2015. The route to protein aggregate superstructures: Particulates and amyloid-like spherulites. *FEBS Lett.* 589 (19PartA):2448–2463. https://doi.org/10.1016/j.febslet.2015.07.006.
14. Becker, S., K. Giller, ..., N. Rezaei-Ghaleh. 2023. Maturation of amyloid β fibrils alters their molecular stability. *Phys. Chem. Chem. Phys.* 25:15099–15103. https://doi.org/10.1039/D3CP01276J.
15. Skamris, T., B. Vestergaard, ..., V. Foderà. 2023. Identifying Biological and Biophysical Features of Different Maturation States of α-Synuclein Amyloid Fibrils. *Methods Mol. Biol.* 2551:321–344. https://doi.org/10.1007/978-1-0716-2597-2_22.
16. Buell, A. K., A. Dhulesia, ..., C. M. Dobson. 2011. Population of nonnative states of lysozyme variants drives amyloid fibril formation. *J. Am. Chem. Soc.* 133:7737–7743. https://doi.org/10.1021/JA109620D.
17. Sivalingam, V., N. L. Prasanna, ..., B. K. Patel. 2016. Wild-type hen egg white lysozyme aggregation in vitro can form self-seeding amyloid conformational variants. *Biophys. Chem.* 219:28–37. https://doi.org/10.1016/j.bpc.2016.09.009.
18. Lara, C., J. Adamcik, ..., R. Mezzenga. 2011. General self-assembly mechanism converting hydrolyzed globular proteins into giant multistranded amyloid ribbons. *Biomacromolecules.* 12:1868–1875. https://doi.org/10.1021/BM200216U.
19. Arnaudov, L. N., and R. De Vries. 2005. Thermally Induced Fibrillar Aggregation of Hen Egg White Lysozyme. *Biophys. J.* 88:515–526. https://doi.org/10.1529/BIOPHYSJ.104.048819.
20. Krebs, M. R., D. K. Wilkins, ..., C. M. Dobson. 2000. Formation and seeding of amyloid fibrils from wild-type hen lysozyme and a peptide fragment from the β-domain. *J. Mol. Biol.* 300:541–549. https://doi.org/10.1006/JMBI.2000.3862.
21. Pullara, F., A. Emanuele, ..., M. U. Palma. 2007. Protein Aggregation/Crystallization and Minor Structural Changes: Universal versus Specific Aspects. *Biophys. J.* 93:3271–3278. https://doi.org/10.1529/BIOPHYSJ.107.110577.
22. Mishra, R., K. Sörgjerd, ..., P. Hammarström. 2007. Lysozyme amyloidogenesis is accelerated by specific nicking and fragmentation but decelerated by intact protein binding and conversion. *J. Mol. Biol.* 366:1029–1044. https://doi.org/10.1016/J.JMB.2006.11.084.
23. Severini, L., K. J. De France, ..., G. Nyström. 2022. Biohybrid Nanocellulose–Lysozyme Amyloid Aerogels via Electrostatic Complexation. *ACS Omega.* 7:578–586. https://doi.org/10.1021/acsomega.1c05069.
24. Ow, S. Y., and D. E. Dunstan. 2013. The effect of concentration, temperature and stirring on hen egg white lysozyme amyloid formation. *Soft Matter.* 9:9692–9701. https://doi.org/10.1039/C3SM51671G.
25. Zein, H. F., I. Alam, ..., T. Sutthibutpong. 2022. Molecular dynamics study on the effects of charged amino acid distribution under low pH condition to the unfolding of hen egg white lysozyme and formation of beta strands. *PLoS One.* 17, e0249742. https://doi.org/10.1371/JOURNAL.PONE.0249742.
26. Yang, L., H. Li, ..., G. Ma. 2019. Amyloid-Based Injectable Hydrogel Derived from Hydrolyzed Hen Egg White Lysozyme. *ACS Omega.* 4:8071–8080. https://doi.org/10.1021/ACSOMEGA.8B03492.
27. Kumari, A., B. Ahmad, and B. Ahmad. 2019. The physical basis of fabrication of amyloid-based hydrogels by lysozyme. *RSC Adv.* 9:37424–37435. https://doi.org/10.1039/C9RA07179B.
28. Nyström, G., L. Roder, ..., R. Mezzenga. 2018. Amyloid Templated Organic–Inorganic Hybrid Aerogels. *Adv. Funct. Mater.* 28:1703609. https://doi.org/10.1002/ADFM.201703609.
29. Alam, I., B. Lertanantawong, ..., P. Asanithi. 2021. Investigating lysozyme amyloid fibrillization by electrochemical impedance spectroscopy for application in lysozyme sensor. *J. Electroanal. Chem.* 901:115799. https://doi.org/10.1016/j.jelechem.2021.115799.
30. Barros, H. R., M. Kokkinopoulou, ..., H. Thérien-Aubin. 2018. Gold nanocolloid–protein interactions and their impact on β-sheet amyloid fibril formation. *RSC Adv.* 8:980–986. https://doi.org/10.1039/C7RA11219J.
31. De Luca, G., D. Fennema Galparsoro, ..., V. Vetri. 2020. Probing ensemble polymorphism and single aggregate structural heterogeneity in insulin amyloid self-assembly. *J. Colloid Interface Sci.* 574:229–240. https://doi.org/10.1016/j.jcis.2020.03.107.
32. Di Carlo, M. G., ..., V. Minicozzi, M. Leone. 2015. Thioflavin T templates Amyloid β(1-40) Conformation and Aggregation pathway. *Biophys. Chem.* 206:1–11.
33. Foderà, V., M. Groenning, ..., M. Leone. 2008. Thioflavin T hydroxylation at basic pH and its effect on amyloid fibril detection. *J. Phys. Chem. B.* 112:15174–15181. https://doi.org/10.1021/JP805560C.
34. D'Amico, M., M. G. Di Carlo, ..., M. Leone. 2012. Thioflavin T promotes Aβ(1-40) amyloid fibrils formation. *J. Phys. Chem. Lett.* 3:1596–1601. https://doi.org/10.1021/JZ300412V.
35. Turoverov K. K., I. M. Kuznetsova, ..., V. N. Uversky. 2007. ThT as an instrument for testing and investigation of amyloid and amyloid-like fibrils. International Conference on Lasers, Applications, and Technologies 2007: Environmental Monitoring and Ecological Applications; Optical Sensors in Biological, Chemical, and Engineering Technologies; and Femtosecond Laser Pulse

Filamentation, 6733; p. 203–213. https://doi.org/10.1117/12.753332

36. Rovnyagina, N. R., G. S. Budylin, ..., E. A. Shirshin. 2020. Fluorescence Lifetime and Intensity of Thioflavin T as Reporters of Different Fibrillation Stages: Insights Obtained from Fluorescence Up-Conversion and Particle Size Distribution Measurements. *Int. J. Mol. Sci.* 21:6169. https://doi.org/10.3390/IJMS21176169.

37. Sulatskaya, A. I., I. M. Kuznetsova, and K. K. Turoverov. 2012. Interaction of thioflavin T with amyloid fibrils: fluorescence quantum yield of bound dye. *J. Phys. Chem. B.* 116:2538–2544. https://doi.org/10.1021/JP2083055.

38. Sulatskaya, A. I., A. A. Maskevich, ..., K. K. Turoverov. 2010. Fluorescence Quantum Yield of Thioflavin T in Rigid Isotropic Solution and Incorporated into the Amyloid Fibrils. *PLoS One.* 5, e15385. https://doi.org/10.1371/JOURNAL.PONE.0015385.

39. Maskevich, A. A., V. I. Stsiapura, ..., K. K. Turoverov. 2007. Spectral Properties of Thioflavin T in Solvents with Different Dielectric Properties and in a Fibril-Incorporated Form. *J. Proteome Res.* 6:1392–1401. https://doi.org/10.1021/pr0605567.

40. Ghosh, R., and D. K. Palit. 2014. Ultrafast Twisting Dynamics of Thioflavin-T: Spectroscopy of the Twisted Intramolecular Charge-Transfer State. *ChemPhysChem.* 15:4126–4131. https://doi.org/10.1002/cphc.201402317.

41. Qin, Z., Y. Sun, ..., G. Ma. 2017. Kinetic Mechanism of Thioflavin T Binding onto the Amyloid Fibril of Hen Egg White Lysozyme. *Langmuir.* 33:5398–5405. https://doi.org/10.1021/ACS.LANGMUIR.7B00221.

42. Amdursky, N., Y. Erez, and D. Huppert. 2012. Molecular Rotors: What Lies Behind the High Sensitivity of the Thioflavin-T Fluorescent Marker. *Acc. Chem. Res.* 45:1548–1557. https://doi.org/10.1021/ar300053p.

43. Stsiapura, V. I., A. A. Maskevich, ..., K. K. Turoverov. 2008. Thioflavin T as a molecular rotor: fluorescent properties of thioflavin T in solvents with different viscosity. *J. Phys. Chem. B.* 112:15893–15902. https://doi.org/10.1021/JP805822C.

44. D'Amico, M., G. Schirò, ..., V. Vetri. 2013. High fluorescence of thioflavin T confined in mesoporous silica xerogels. *Langmuir.* 29:10238–10246. https://doi.org/10.1021/LA402406G.

45. Sulatskaya, A. I., N. P. Rodina, ..., K. K. Turoverov. 2018. Investigation of α-Synuclein Amyloid Fibrils Using the Fluorescent Probe Thioflavin T. *Int. J. Mol. Sci.* 19:2486. https://doi.org/10.3390/IJMS19092486.

46. Lindberg, D. J., M. S. Wranne, ..., E. K. Orner. 2015. Steady-state and time-resolved Thioflavin-T fluorescence can report on morphological differences in amyloid fibrils formed by Ab(1-40) and Ab(1-42). *Biochem. Biophys. Res. Commun.* 458:418–423. https://doi.org/10.1016/j.bbrc.2015.01.132.

47. Sancataldo, G., S. Anselmo, and V. Vetri. 2020. Phasor-FLIM analysis of Thioflavin T self-quenching in Concanavalin amyloid fibrils. *Microsc. Res. Tech.* 83:811–816. https://doi.org/10.1002/jemt.23472.

48. Pedersen, J. S., D. Dikov, ..., D. E. Otzen. 2006. The Changing Face of Glucagon Fibrillation: Structural Polymorphism and Conformational Imprinting. *J. Mol. Biol.* 355:501–523. https://doi.org/10.1016/J.JMB.2005.09.100.

49. Kuimova, M. K. 2012. Mapping viscosity in cells using molecular rotors14. pubs.rsc.org, pp. 12671–12686. https://pubs.rsc.org/en/content/articlehtml/2012/cp/c2cp41674c. (Accessed 18 July 2023), Accessed.

50. Sidhu, A., J. Vaneyck, ..., V. Subramaniam. 2018. Polymorph-specific distribution of binding sites determines thioflavin-T fluorescence intensity in α-synuclein fibrils. *Amyloid.* 25:189–196. https://doi.org/10.1080/13506129.2018.1517736.

51. Thompson, A. J., T. W. Herling, ..., M. K. Kuimova. 2015. Molecular Rotors Provide Insights into Microscopic Structural Changes during Protein Aggregation. *J. Phys. Chem. B.* 119:10170–10179. https://doi.org/10.1021/ACS.JPCB.5B05099.

52. Mohanty, J., S. D. Choudhury, ..., A. C. Bhasikuttan. 2012. Early detection of insulin fibrillation: a fluorescence lifetime assay to probe the pre-fibrillar regime. *Chem. Commun.* 48:2403–2405. https://doi.org/10.1039/C2CC16974F.

53. Morando, M. A., F. Venturella, ..., C. Alfano. 2022. Solution structure of recombinant Pvfp-5β reveals insights into mussel adhesion. *Commun. Biol.* 5:739. https://doi.org/10.1038/S42003-022-03699-W.

54. Rodina, N. P., M. I. Sulatsky, ..., K. K. Turoverov. 2017. Photophysical Properties of Fluorescent Probe Thioflavin T in Crowded Milieu. *Journal of Spectroscopy.* 2017:1–10. https://doi.org/10.1155/2017/2365746.

55. Malacrida, L., S. Ranjit, ..., E. Gratton. 2021. The Phasor Plot: A Universal Circle to Advance Fluorescence Lifetime Analysis and Interpretation. *Annu. Rev. Biophys.* 50:575–593. https://doi.org/10.1146/ANNUREV-BIOPHYS-062920-063631.

56. Ranjit, S., L. Malacrida, ..., E. Gratton. 2018. Fit-free analysis of fluorescence lifetime imaging data using the phasor approach. *Nat. Protoc.* 13:1979–2004. https://doi.org/10.1038/s41596-018-0026-5.

57. Ranjit, S., L. Malacrida, and E. Gratton. 2018. Differences between FLIM phasor analyses for data collected with the Becker and Hickl SPC830 card and with the FLIMbox card. *Microsc. Res. Tech.* 81:980–989. https://doi.org/10.1002/JEMT.23061.

58. Michalet, X. 2021. Continuous and discrete phasor analysis of binned or time-gated periodic decays. *AIP Adv.* 11:035331. https://doi.org/10.1063/5.0027834.

59. Coucke, Q., N. Parveen, ..., J. Hendrix. 2023. Particle-based phasor-FLIM-FRET resolves protein-protein interactions inside single viral particles. *Biophys. Rep. (N Y).* 3:100122. https://doi.org/10.1016/j.bpr.2023.100122.

60. Digman, M. A., V. R. Caiolfa, ..., E. Gratton. 2008. The phasor approach to fluorescence lifetime imaging analysis. *Biophys. J.* 94:L14–L16. https://doi.org/10.1529/biophysj.107.120154.

61. Data Tables | Fluorescence Lifetime Standards | ISS. https://iss.com/resources/reference/data_tables/FL_LifetimeStandards.html.

62. Biancalana, M., and S. Koide. 2010. Molecular Mechanism of Thioflavin-T Binding to Amyloid Fibrils. *Biochim. Biophys. Acta.* 1804:1405–1412. https://doi.org/10.1016/j.bbapap.2010.04.001.

63. LeVine, H. 1999. Quantification of β-sheet amyloid fibril structures with thioflavin T. *Methods Enzymol.* 309:274–284. https://doi.org/10.1016/S0076-6879(99)09020-5.

64. Barth, A. 2007. Infrared spectroscopy of proteins. *Biochim. Biophys. Acta.* 1767:1073–1101. https://doi.org/10.1016/J.BBABIO.2007.06.004.

65. Dong, A., J. Matsuura, ..., J. F. Carpenter. 1996. Infrared and Circular Dichroism Spectroscopic Characterization of Structural Differences between β-Lactoglobulin A and B. *Biochemistry.* 35:1450–1457. https://doi.org/10.1021/BI9518104.

66. Kelly, S. M., and N. C. Price. 1997. The application of circular dichroism to studies of protein folding and unfolding. *Biochim. Biophys. Acta.* 1338:161–185. https://doi.org/10.1016/S0167-4838(96)00190-2.

67. Greenfield, N. J. 2006. Using circular dichroism spectra to estimate protein secondary structure. *Nat. Protoc.* 1:2876–2890. https://doi.org/10.1038/nprot.2006.202.

68. Morozova-Roche, L. A., J. A. Jones, ..., C. M. Dobson. 1999. Independent Nucleation and Heterogeneous Assembly of Structure During Folding of Equine Lysozyme. *J. Mol. Biol.* 289:1055–1073. https://doi.org/10.1006/JMBI.1999.2741.

69. Foderà, V., V. Vetri, ..., B. Vestergaard. 2014. Observation of the early structural changes leading to the formation of protein superstructures. *J. Phys. Chem. Lett.* 5:3254–3258. https://doi.org/10.1021/JZ501614E.

70. Piccirilli, F., G. Schirò, …, V. Militello. 2015. Decoding vibrational states of Concanavalin A amyloid fibrils. *Biophys. Chem.* 199:17–24. https://doi.org/10.1016/j.bpc.2015.02.007.

71. Zandomeneghi, G., M. R. H. Krebs, …, M. Fändrich. 2004. FTIR reveals structural differences between native β-sheet proteins and amyloid fibrils. *Protein Sci.* 13:3314–3321. https://doi.org/10.1110/ps.041024904.

72. Usoltsev, D., V. Sitnikova, …, M. Uspenskaya. 2019. Systematic FTIR Spectroscopy Study of the Secondary Structure Changes in Human Serum Albumin under Various Denaturation Conditions. *Biomolecules.* 9:359. https://doi.org/10.3390/biom9080359.

73. Jackson, M., and H. H. Mantsch. 1991. Protein secondary structure from FT-IR spectroscopy: correlation with dihedral angles from three-dimensional Ramachandran plots. *Can. J. Chem.* 69:1639–1642. https://doi.org/10.1139/V91-240.

74. Arosio, P., T. P. J. Knowles, and S. Linse. 2015. On the lag phase in amyloid fibril formation. *Phys. Chem. Chem. Phys.* 17:7606–7618. https://doi.org/10.1039/C4CP05563B.

75. Sulatskaya, A. I., A. V. Lavysh, …, K. K. Turoverov. 2017. Thioflavin T fluoresces as excimer in highly concentrated aqueous solutions and as monomer being incorporated in amyloid fibrils. *Sci. Rep.* 7:2146. https://doi.org/10.1038/s41598-017-02237-7.

76. Ranjit, S., R. Datta, …, E. Gratton. 2019. Multicomponent Analysis of Phasor Plot in a Single Pixel to Calculate Changes of Metabolic Trajectory in Biological Systems. *J. Phys. Chem. A.* 123:9865–9873. https://doi.org/10.1021/ACS.JPCA.9B07880.

77. Singh, P. K., M. Kumbhakar, …, S. Nath. 2009. Ultrafast torsional dynamics of protein binding dye thioflavin-T in nanoconfined water pool. *J. Phys. Chem. B.* 113:8532–8538. https://doi.org/10.1021/JP902207K.

78. Lindgren, M., K. Sö, and P. Hammarströ. 2005. Detection and Characterization of Aggregates, Prefibrillar Amyloidogenic Oligomers, and Protofibrils Using Fluorescence Spectroscopy. *Biophys. J.* 88:4200–4212. https://doi.org/10.1529/biophysj.104.049700.

79. Fennema Galparsoro, D.…, 2021. Conformational Transitions upon Maturation Rule Surface and pH-Responsiveness of α-Lactalbumin Microparticulates. *ACS Appl. Bio Mater.* 4:1876–1887. https://doi.org/10.1021/acsabm.0c01541.

Biophysical Reports, Volume *4*

Supplemental information

Deciphering amyloid fibril molecular maturation through FLIM-phasor analysis of thioflavin T

Sara Anselmo, Giuseppe Sancataldo, and Valeria Vetri

Deciphering Amyloid Fibril Molecular Maturation through FLIM-Phasor Analysis of Thioflavin T

Sara Anselmo[1,]*, Giuseppe Sancataldo[1,] and Valeria Vetri[1]

[1]Dipartimento di Fisica e Chimica – Emilio Segré, Università degli Studi di Palermo, Viale delle Scienze ed. 18, 90128 Palermo, Italy

* Corresponding author:
sara.anselmo@unipa.it

Supporting Material

ThT-stained lysozyme sample after 30 min of incubation

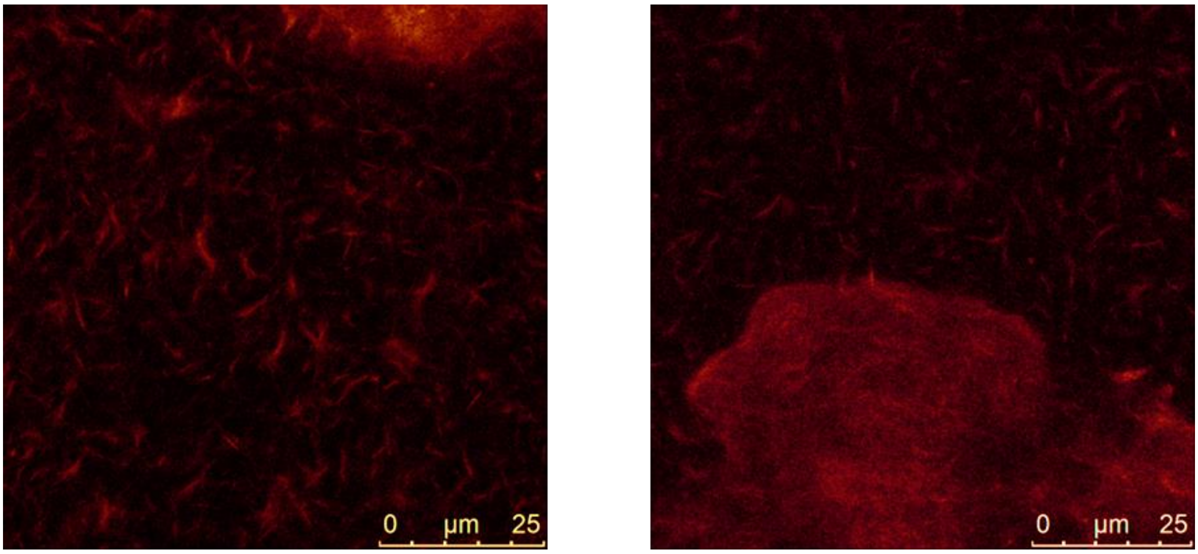

SM.1 1024x1024 pixels representative confocal images of 40 mg/ml HEWL pH 2.0 incubated at 90°C with magnetic stirring at 300 rpm for 30 min and stained with 60 μM ThT (λ_{exc}= 470 nm, emission range 485 nm -600 nm).

Ovoid-shaped lysozyme aggregates formed after 60 miN of incubation

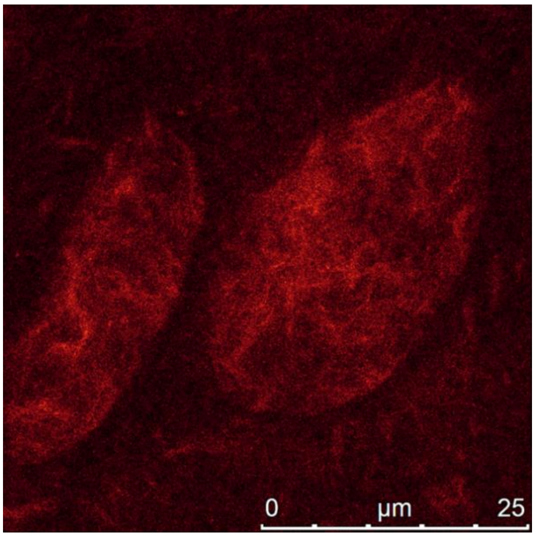

SM.2 1024x1024 pixels representative confocal images of 40 mg/ml HEWL pH 2 incubated at 90°C with magnetic stirring at 300 rpm for 60 min and stained with 60 μM ThT (λ_{exc}= 470 nm, emission range 485 nm -600 nm).
